# Procaspase-3-activating compound 1 stabilizes hypoxia-inducible factor 1α and induces DNA damage by sequestering ferrous iron

**DOI:** 10.1038/s41419-018-1038-3

**Published:** 2018-10-04

**Authors:** Feifei Li, Aili Wei, Lijuan Bu, Long Long, Wei Chen, Chen Wang, Changqi Zhao, Lili Wang

**Affiliations:** 1 Beijing Institute of Pharmacology and Toxicology, State Key Laboratory of Toxicology and Medical Countermeasures, Beijing, 100850 China; 2 State Key Laboratory of Functions and Applications of Medicinal Plants, Guizhou Medical University, Guiyang, 550014 China; 3 Key Laboratory of Cell Proliferation and Regulation Biology, Beijing Normal University, Beijing, 100875 China

## Abstract

Procaspase-3-activating compound 1 (PAC-1) induces procaspase-3 activation via zinc chelation. However, whether PAC-1 employs other mechanisms remains unknown. Here we systematically screened for potent PAC-1 targets using 29 enhanced green fluorescent protein-labeled reporter cell lines and identified hypoxia-inducible factor 1α (HIF1α) and RAD51 pathways as PAC-1 targets. These results were verified in HepG2 cells and two other cancer cell lines. Mechanistically, PAC-1 specifically blocked HIF1α hydroxylation and upregulated HIF1α target genes. In addition, DNA damage, G_1_/S cell cycle arrest, and the inhibition of DNA synthesis were induced following PAC-1 administration. Interestingly, by using ferrozine-iron sequestration and iron titration assays, we uncovered the iron sequestering capacity of PAC-1. Additionally, the expression levels of iron shortage-related genes were also increased in PAC-1-treated cells, and iron (II) supplementation reversed all of the observed cellular responses. Thus, our results indicate that PAC-1 induces HIF1α stabilization and DNA damage by sequestering ferrous iron.

## Introduction

Escaping apoptosis represents one of the hallmarks of cancer, and the induction of apoptotic cell death is a rational anticancer strategy. However, the dysregulation of apoptotic mechanisms and overexpression of anti-apoptotic proteins often limit the efficacy of apoptosis-inducing agents^1^. The discovery of procaspase-3-activating compound 1 (PAC-1) may overcome this limitation. By activating procaspase-3 to generate caspase-3, the main apoptosis effector, PAC-1 bypasses the complex upstream pro-apoptotic signaling cascades and directly induces apoptotic cell death^2^. Procaspase-3 activators have since attracted much attention, and a series of compounds targeting procaspase-3 have been discovered^3–7^. However, the first report describing PAC-1 did not address the mechanisms underlying procaspase-3 activation, and these still remain unclear to date^8^. Hergenrother and co-workers reported that PAC-1 activates procaspase-3 by chelating the zinc ions required for its activity^9^. Although this mechanism has been widely accepted, it might not account for the full function of PAC-1. Moreover, the antitumor effect of PAC-1 has not been so far validated in humans.

In this study, we aimed to further elucidate the mechanisms underlying PAC-1 function. To this end, we analyzed the effects of PAC-1 on 29 pathways/proteins using enhanced green fluorescent protein (EGFP)-labeled reporter cell lines (Table 1). We then further investigated the mechanisms of PAC-1 on the hypoxic response and DNA damage in cancer cells.

**Table 1:** The main information of signal pathways used in screening

Targets or pathways	Cell lines	Compound incubation time	Mode of analysis	*Z*′ factor
AKT	Akt1-EGFP_CHO	4 min	Membrane translocation^a^	0.68 ± 0.09
AR	AR-EGFP_U2OS	6 h	Nuclear foci formation^b^	0.63 ± 0.02
ATF6	ATF6-EGFP_U2OS	5 h	Nuclear translocation^c^	0.58 ± 0.09
BTK	Btk-EGFP_CHO	4 min	Membrane translocation	0.74 ± 0.11
CBR1	CBR1-EGFP_U2OS	2 h	Internalization^d^	0.55 ± 0.21
ERα	ERα-EGFP_U2OS	20 h	Nuclear foci formation	0.67 ± 0.08
FSHR	rFSHR-EGFP_U2OS	2 h	Internalization	0.57 ± 0.06
GLP-1R	GLP1R-EGFP_U2OS	1 h	Internalization	0.76 ± 0.04
GLUT4	GLUT4-EGFP_CHO	5 min	Membrane translocation	0.49 ± 0.12
Hif1α	Hif1α-EGFP_CHO	3 h	Nuclear accumulation^e^	0.55 ± 0.10
JAK/STAT1	STAT1-EGFP_U2OS	1 h	Nuclear translocation	0.85 ± 0.05
JAK/STAT3	STAT3-EGFP_BHK	0.5 h	Nuclear translocation	0.64 ± 0.08
M3/NFATc1	M3:NFATc1-EGFP_U2OS	20 min	Nuclear translocation	0.74 ± 0.10
NF-κB	p65-EGFP_CHO	40 min	Nuclear translocation	0.50 ± 0.12
p27 ubiquitination	SCF-Skp2 E3 Ligase: p27-EGFP_HeLa	24 h	Nuclear translocation	0.82 ± 0.08
p38 MAPK	MAPKAPk2-EGFP_BHK	1.5 h	Nuclear translocation	0.56 ± 0.09
p53 ubiquitination	E6-AP: p53-EGFP_HeLa	24 h	Nuclear accumulation	0.81 ± 0.05
p53-Hdm2	p53-Hdm2-EGFP_CHO	2 h	Nuclear translocation	0.63 ± 0.01
PI3K-FKHR	FKHR-EGFP_U2OS	1 h	Nuclear translocation	0.66 ± 0.05
PI3K-FKHRL1	FKHRL1-EGFP_U2OS	1 h	Nuclear translocation	0.59 ± 0.07
PI3K-Foxo4	Foxo4-EGFP_U2OS	1 h	Nuclear translocation	0.58 ± 0.10
PI3K-PDK1	PDK1-EGFP_CHO	2 min	Membrane translocation	0.73 ± 0.12
PKA	PKAcat-EGFP_CHO	5 min	Foci formation in cytoplasm^f^	0.67 ± 0.08
PKCβ	PKC β-EGFP_U2OS	0 min	Membrane translocation	0.28 ± 0.05
PR	PR-EGFP_U2OS	30 min	Nuclear foci formation	0.43 ± 0.05
Rad51	Rad51-EGFP_SW480	24 h	Nuclear foci formation^c^	0.73 ± 0.10
Ras-Raf	Raf-EGFP_CHO	5 min	Membrane translocation	0.54 ± 0.09
S1P1	S1P_1_-EGFP_U2OS	1 h	Internalization^e^	0.83 ± 0.03
TGFβ-Smad2	Smad2-EGFP_CHO	1 h	Nuclear translocation	0.52 ± 0.14

^a^Membrane translocation indicate EGFP-labeled proteins translocate from the cytoplasm onto the cell membrane. This analysis mode analyses the amount of EGFP patch on the cell membrane

^b^Nuclear foci formation indicate EGFP-labeled proteins form foci inside the nucleus. This analysis mode analyses the amount of EGFP foci inside the nucleus

^c^Nuclear translocation indicate EGFP-labeled proteins translocate from cytoplasm into the nucleus or simply accumulation in the nucleus. This analysis mode analyses the EGFP intensity in the nucleus

^d^Internalization indicate EGFP-labeled proteins translocate from cell membrane into cytoplasm

^e^Nuclear accumulation indicate EGFP-labeled proteins appears and accumulation in the nuclear after positive compound treatment

^f^Foci formation in cytoplasm means translocation of PKAcat-EGFP from cytoplasmic aggregates to a uniform cytoplasmic localization

## Materials and methods

### Cell culture

Fluorescently labeled reporter cell lines were purchased from Thermo Fisher Scientific (Waltham, MA), except for SMAD2-EGFP_CHO, MAPKAPK2-EGFP_BHK, and AKT1-EGFP_CHO, which were purchased from GE Healthcare (Fairfield, CT). All cell lines were cultured following the manufacturer’s recommendation. The human hepatic carcinoma cell line HepG2 was purchased from the Type Culture Collection of the Chinese Academy of Sciences (Shanghai, China), and cultured in RPMI 1640 medium supplied with 10% fetal bovine serum.

### Compounds and reagents

PAC-1, BP (2,2′-dipyridyl, a well-known iron (II) chelator and chemical hypoxia mimic), MG132, and ferrozine were purchased from Sigma-Aldrich (St. Louis, MO). Other reagents and all cell culture media were purchased from Thermo Fisher unless specifically stated. Primary antibodies targeting pH2AX (cat. no. H10292) and BrdU and labeled secondary antibodies were obtained from Life Technologies (Beverly, MA). Primary antibodies targeting hypoxia-inducible factor 1α (HIF1α; cat. no. ab16066) and Pro564 hydroxylated HIF1α (cat. no. 3434) were purchased from Abcam (Cambridge, MA) and Cell Signaling Technology (Danvers, MA), respectively.

### Immunofluorescence staining for high-content analysis

Immunofluorescence staining for pH2AX, HIF1α, HIF1α-OH, and BrdU was conducted as described below. Briefly, cells were plated in Corning 3603 plates (black-wall, clear bottom 96-well plates; cat. no. 3603; Corning, Corning, NY), treated with different compounds, fixed with 4% formaldehyde, and washed twice with 1× phosphate-buffered saline (PBS). After permeabilizing the cell membranes using 0.1% Triton X-100 and blocking with 10% bovine serum albumin, target proteins were visualized using primary antibodies and fluorescently labeled secondary antibodies. Cell nuclei were labeled with 1 μM Hoechst 33342 and subjected to image acquisition on an high-content analysis (HCA) platform.

### HCS for signaling pathways or target proteins

Twenty-nine genetically modified reporter cell lines were used in cell-based signaling pathway or target protein screening. For each cell line, we followed the screening procedures recommended by the manufacturer; key information is briefly described in Table 1. The concentrations of PAC-1 used for screening were 3 and 30 μM. Detailed information regarding the RAD51 and HIF1α assays is provided below. In order to display the screening results more intuitively, heat map was made basing on preliminary screening data using MeV software.

HIF1α-EGFP_CHO cells (CHO cells stably expressing the HIF1α-EGFP fusion protein) or RAD51-EGFP_SW480 cells (SW480 cells stably expressing the RAD51-EGFP fusion protein) were seeded in Corning 3603 plates at a density of 8 × 10^3^ cells/well for 24 or 48 h to allow adhesion. Then, different concentrations of PAC-1 were added and the plates were incubated for 3 or 24 h. Finally, cells were fixed with 4% formaldehyde, and the nuclei were dyed with 1 μM Hoechst 33342 for 30 min at 37 °C. The cells were then subjected to high-content image acquisition and analysis using an IN CELL Analyzer 1000 platform. The activity of PAC-1 in HIF1α pathway assays was expressed as the activation rate relative to that of the positive compound (100 μM BP) and negative control (0.2% dimethylsulfoxide [DMSO]). The activity of PAC-1 in RAD51 pathway assays was expressed as the activation rate relative to that of the positive compound (10 μM camptothecin) and negative control (0.2% DMSO) for screening and as the fold change of RAD51 intensity relative to the negative control (0.2% DMSO) for EC_50_ testing.

### BrdU incorporation for HCA

BrdU incorporation assays were performed using Click-iT EdU HCS Assays (Invitrogen, Thermo Fisher Scientific). Briefly, HepG2/U2OS cells were plated in Corning 3603 plates at a density of 1 × 10^4^ or 9 × 10_3_ cells/well, respectively, and cultured for 24 h before exposure to PAC-1 for 3 h. Next, 100 μL BrdU (20 μM) was added, and cells were incubated for another 4 h to allow BrdU incorporation into the newly synthesized DNA. Cells were then fixed, and the incorporated BrdU was labeled with Alexa Fluor 555. Cell nuclei were dyed with HCS Nuclear Mask Blue stain. Finally, cells were used for high-content image acquisition and analysis using the IN CELL platform. The inhibition of DNA synthesis by PAC-1 was expressed as the rate of change in BrdU-positive cells and the mean fluorescence intensity of BrdU-Alexa Fluor in each cell.

### High-content image acquisition and analysis

Fluorescence images were all acquired with an IN CELL Analyzer 1000/2000 (GE Healthcare) under a ×10 objective lens except that in supplement Fig. 6, as reported previously^10,11^. At least five fields and more than 500 cells/well were analyzed using the Multi Target Analysis Module of the IN CELL Analyzer Workstation 3.5. Each treatment was repeated in triplicate, and each experiment was repeated at least three times to ensure the robustness and reliability of the results.

### Flow cytometric cell cycle analysis

Flow cytometric cell cycle analysis was conducted according to generally accepted procedures^12^. Briefly, HepG2 cells were harvested and fixed with 70% ethanol overnight after 24 h of PAC-1 exposure. Cells were then washed three times with PBS and treated with 50 μg/mL RNase at 37 °C for 30 min. Finally, cells were labeled with propidium iodide and analyzed with a FACSCalibur instrument (BD Biosciences, MA, USA).

### Quantitative polymerase chain reaction

HepG2 cells were exposed to the compounds under normoxic or hypoxic conditions (1% oxygen) for the indicated times. Total RNA was extracted using a BioTeke Total RNA Extraction Kit (cat. no. RP1201; BioTeke, Beijing, China). RNA was then reverse transcribed into single-stranded cDNA using TransScript SuperMix (cat. no. AT311-03; TransGen, Beijing, China). The resulting cDNA was used as a template for quantitative polymerase chain reaction (qPCR). The primers used in this study are listed in Table 2. qPCR was conducted using the ABI PRISM 7900 sequence detection system (Applied Biosystems, Foster City, CA), as previously described^13^. The relative change in mRNA levels was determined using the 2^−ΔΔCT^ method, and β-actin was used as the internal standard.

**Table 2:** The primers used in quantitative real-time PCR

	Sense	Antisense
Hif1α	5′-CTCAAAGTCGGACAGCCTCA-3′	5′-CCCTGCAGTAGGTTTCTGCT-3′
VEGFA	5′-CTTGCCTTGCTGCTCTACC-3′	5′-CACACAGGATGGCTTGAAG-3′
EPO	5′-ACCAACATTGCTTGTGCCAC-3′	5′-TCTGAATGCTTCCTGCTCTGG-3′
TfR1	5′-ACTTGCCCAGATGTTCTCAG-3′	5′-GTATCCCTCTAGCCATTCAGTG-3′
DMT1	5′-TGTTTGATTGCACTGGGCATG-3′	5′-CTGTCAGCAGGCCTTTAGAG-3′
β-actin	5′-TAAAGACCTCTATGCCAACACAGT-3′	5′-CACGATGGAGGGGCCGGACTCATC-3′

### Western blotting

Cells were lysed after the indicated treatments. Approximately, 5 mg of total protein for each sample were separated by sodium dodecyl sulfate polyacrylamide gel electrophoresis on 10% gels and then transferred to polyvinylidene difluoride membranes. The membranes were blocked with 5% fat-free milk and incubated with appropriate primary antibodies at 4 °C overnight, followed by incubation with horseradish peroxidase-conjugated secondary antibodies at room temperature for 1 h. The immunostaining signals were visualized using the ECL plus reagent (Applygen, Beijing, China), imaged, and analyzed with an Alpha Imager 5500 (Alpha Innotech, San Leandro, CA).

### Ferrozine-iron (II) chelation assay

The iron (II)-chelating activity of PAC-1 was assessed as previously described^17^, under nitrogen. Briefly, 19.6 mL double-distilled water (DDW) and 400 μL of 0.2 mM FeCl_2_ (final concentration 4 μM) were premixed, and an aliquot of 198 μL was added into each well of a 96-well plate (Corning 3599). Next, 2 μL/well of 100× drugs in DMSO were added. The plate was shaken on a microplate shaker for 5 min and incubated for 30 min to allow for equilibration. Next, ferrozine (final concentration 200 μM) was added, and the plate was shaken on a microplate shaker for 10 min. Finally, the absorbance at 550 nm was scanned using a SpectraMax M5 instrument (Molecular Devices, Eugene, OR). The signal for control group 1, which contained only DDW and ferrozine, was set as 100% iron chelation, whereas the signal for control group 2, which contained DDW, FeCl_2_, and ferrozine, was set as 0% chelation. The chelation rate for the treatment groups was calculated as follows:

$${{\rm CR}} = \left( {{{\rm OD}}_{{{\rm treatment}}}- {{\rm OD}}_{{{\rm control2}}}} \right)/\left( {{{\rm OD}}_{{{\rm control1}}}- {{\rm OD}}_{{{\rm control2}}}} \right) \times 100\%$$

### Iron (II) titration assay

The titration of PAC-1 using Fe(II) or Zn(II) was conducted as previously described^9^ in buffer A (50 mM HEPES, 100 mM KNO_3_, pH 7.2) under nitrogen. Briefly, the absorbance spectrum of PAC-1 in 3 mL buffer A (final concentration 50 μM) from 230 to 500 nm was scanned using a SpectraMax M5 (Molecular Devices). Then, 1.5 μL of 10 mM FeCl_2_ (or ZnSO_4_) were added to the system, and the absorbance spectrum was scanned again. The last two processes were repeated 15 times until the concentration of FeCl_2_ in the system reached 75 μM.

### Iron supplementation assays

The effects of iron supplementation on the PAC-1-induced formation of RAD51 foci, H2AX phosphorylation, and HIF1α accumulation were examined. PAC-1 and FeCl_2_ (or ZnSO_4_ in HIF1α accumulation assays) were added sequentially to the cell culture medium and incubated for the indicated duration.

The effects of iron supplementation on the antiproliferative activity of PAC-1 were also examined. HepG2 cells plated in 96-well plates were exposed to PAC-1 or PAC-1 plus FeCl_2_ for 48 h. The nuclei were then labeled with 1 μM Hoechst 33342 for 30 min before image acquisition using an IN CELL Analyzer 1000, and the cell counts for the different treatment groups were analyzed.

### Iron rescue assay

HepG2 cells plated in 96-well plates were exposed to 30 μM PAC-1 for 24 h. At 0, 0.5, 1, 2, 4, or 6 h after PAC-1 addition, 30 μM FeCl_2_ was added. After 24 h of PAC-1 treatment, nuclei were labeled with 1 μM Hoechst 33342, and the cell counts for the different treatment groups were analyzed using an IN CELL Analyzer 1000.

### Data processing

All data were processed with Microsoft Excel (2010) and reported as means ± standard deviations. Concentration–effect curves were drawn, and the EC_50_ values were calculated using the GraphPad Prism 5 software. The statistical analysis was conducted using a one-way analysis of variance in SPSS 13.0 and Tukey tests for post hoc multiple comparisons. Differences with *P* values of <0.05 were considered significant.

## Results

### Screening of multiple signaling pathways

**Fig. 1:**
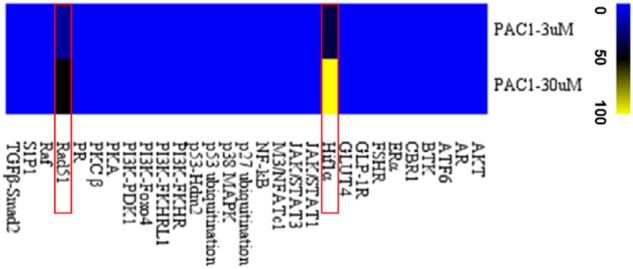
Heat map of the PAC-1 screening results for multiple signaling pathways or targets. The activity of PAC-1 in pathway assays was expressed as the activation rate relative to the positive compound (100 μM BP in the HIF1α pathway and 10 μM camptothecin in the RAD51 pathway) and negative control (0.2% DMSO)

To comprehensively investigate the effects of PAC-1 on multiple signaling pathways or target proteins, an unbiased screening assay was conducted using HCA and 29 EGFP-labeled reporter cell lines representing different signaling pathways or targets. The *Z′* factor for almost all assays was >0.5 (Table 1), indicating that these cellular models were eligible for high-content screening (HCS) and that the screening system was reliable. As shown in Fig. 1, a 3 or 30 μM concentration of PAC-1 did not affect the majority of signaling pathways or target proteins, except for the RAD51 and HIF1α pathways. In the two positive cell lines, PAC-1 showed significant concentration-dependent effects, including the nuclear translocation of HIF1α and the formation of RAD51 nuclear foci. Moreover, a 30 μM dose of PAC-1 induced a similar effect to the maximum effect observed with 100 μM of BP in HIF1α assays and approximately half that observed in the presence of 10 μM of camptothecin in RAD51 assays. These screening results indicate that PAC-1 selectively acts on the HIF1α and RAD51 signaling pathways.

### PAC-1 induces HIF1α stabilization under normoxic conditions

**Fig. 2:**
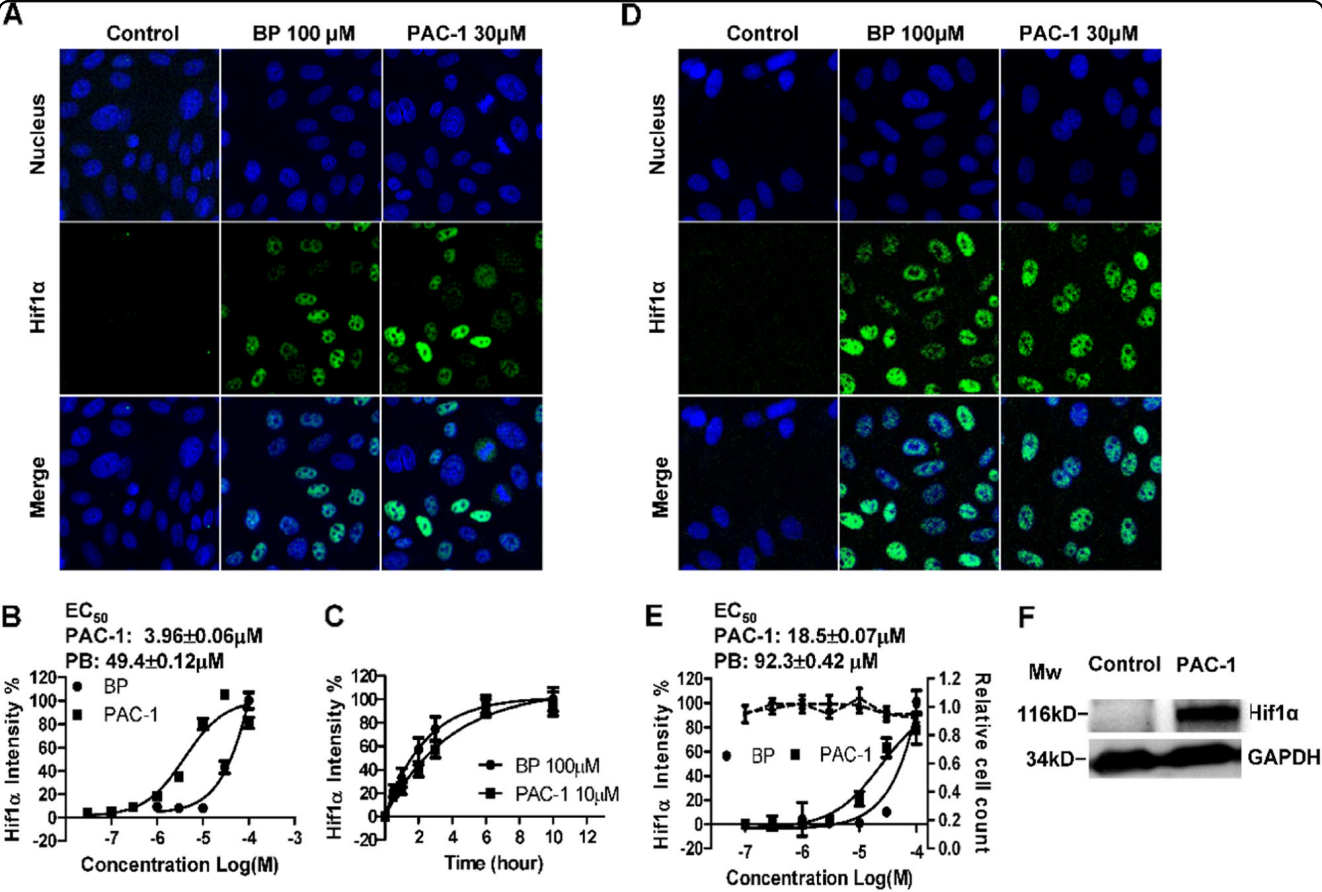
PAC-1 induces the stabilization of HIF1α under normoxic conditions. HIF1α-EGFP_CHO cells were treated with the indicated drugs for 3 h before fixation, acquisition of fluorescence images (**a**), and quantitative analysis (**b**). **c** Time course of HIF1α accumulation in HIF1α-EGFP_CHO cells. **d**–**f** HepG2 cells were treated with the indicated drugs for 3 h, and HIF1α levels were detected using specific antibodies. **d** Fluorescence images of stabilized HIF1α in HepG2 cells. **e** Concentration-dependent accumulation of HIF1α in HepG2 cells (black solid line) and changes in cell counts (black dashed line). **f** Western blotting of HIF1α accumulation in HepG2 cells. Data represent means ± SEs of three independent experiments

To further examine the effects of PAC-1 on HIF1α in HIF1α-EGFP_CHO cells, a series of concentrations of PAC-1 and the chemical hypoxia mimic BP (the well-known iron (II) chelator) were used, and the time-dependent effects following treatment with PAC-1 or BP were evaluated. As shown in Fig. 2a, considerable HIF1α fluorescence was observed in the nucleus after 3 h of BP or PAC-1 treatment when compared with that in the untreated control group. A quantitative analysis of the HIF1α fluorescence intensity showed that PAC-1 induced HIF1α accumulation in a concentration-dependent manner (Fig. 2b). The calculated EC_50_ value was 3.96 μM, which was lower than that of BP. The kinetics of HIF1α accumulation indicated that PAC-1 could induce HIF1α accumulation after only 0.5 h of PAC-1 exposure and that the HIF1α protein levels continued to increase until reaching a plateau after about 6 h of PAC-1 exposure, similar to our BP results (Fig. 2c). Moreover, this property of PAC-1 was not restricted to genetically modified HIF1α-EGFP reporter cell lines, as a concentration-dependent increase in HIF1α protein levels was also observed in PAC-1-treated HepG2 cells (Fig. 2d–f), with an EC_50_ of 18.5 ± 0.07 μM. Additionally, a 3 h PAC-1 treatment had no effect on cell counts and nuclear shape at the corresponding concentrations (Fig. 2e). These results indicate that PAC-1 induces HIF1α accumulation under normoxic conditions, independent of cellular cytotoxicity.

### PAC-1 induces the accumulation of functional HIF1α by inhibiting HIF1α hydroxylation

**Fig. 3:**
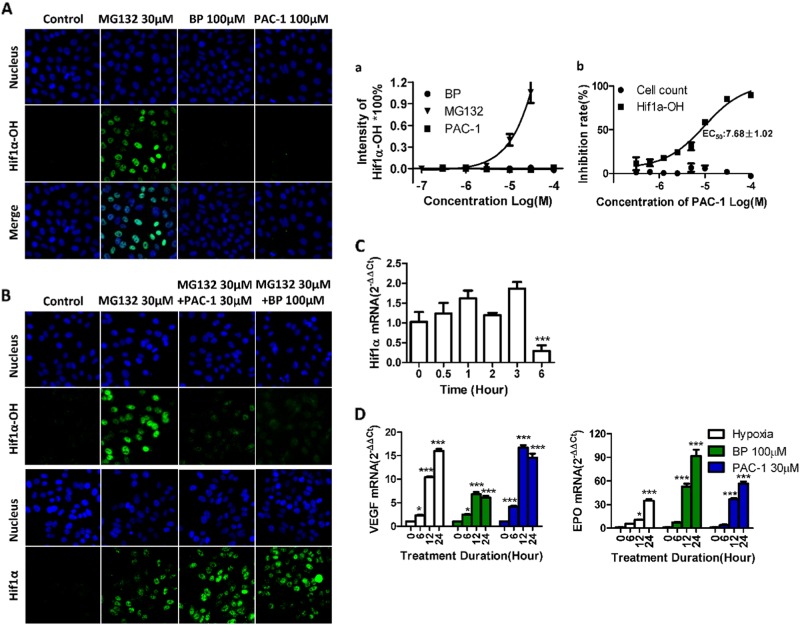
Characteristics of the PAC-1-induced HIF1α. **A** HepG2 cells were exposed to different compounds for 3 h before immunofluorescence labeling of hydroxylated HIF1α using Pro564 hydroxylated HIF1α-specific antibodies and quantitative analysis (**a**). (**B**) HepG2 cells were exposed to 30 μM MG132 with BP or different concentrations of PAC-1 for 3 h before immunofluorescence labeling of hydroxylated HIF1α or HIF1α using Pro564 hydroxylated HIF1α or HIF1α-specific antibodies and quantitative analysis (**b**). **C** HepG2 cells were exposed to 30 μM MG132 with 30 μM PAC-1 and 100 μM BP for 3 h before immunofluorescence labeling of HIF1α. **D** Changes in HIF1α mRNA levels in HepG2 cells exposed to 30 μM PAC-1. ***P < 0.001 compared with untreated cells. **D** mRNA levels of HIF1α target genes after HepG2 cells were exposed to compounds or cultured under hypoxic conditions (1% oxygen level) for the indicated times. Data represent means ± SEs of three independent experiments. *P < 0.05; ***P < 0.001; compared with the 0 h treatment group

HIF1α has a short half-life and is constitutively expressed in cells. Additionally, this protein undergoes hydroxylation, ubiquitination, and proteasomal degradation after translation under normal oxygen levels^14^. Because HIF1α was induced by PAC-1 under normoxic conditions, we first examined the hydroxylation status of HIF1α using specific antibodies targeting proline 564 (Pro564) hydroxylated HIF1α. Furthermore, treatment with the ubiquitin proteasome inhibitor MG132, which was used as a positive control because the ablation of proteasomal activity will result in the stabilization of hydroxylated HIF1α. Conversely, the iron chelator BP was used as a negative control because BP induces HIF1α accumulation by sequestering iron and blocking the activity of prolyl hydroxylase domain protein 2 (PHD2), the enzyme responsible for HIF1α hydroxylation. As shown in Fig. 3A, a concentration-dependent accumulation of hydroxylated HIF1α was detected in MG132-treated cells, whereas no fluorescent signal was detectable in PAC-1- and BP-treated cells. These findings suggest that the hydroxylation of HIF1α was also blocked by PAC-1. To further confirm these specific effects on hydroxylation, we investigated the impact of PAC-1 on HIF1α-OH and HIF1α respectively following treatment with MG132, as shown in Fig. 3B, b, notably, PAC-1 reduced HIF1α-OH levels in a concentration-dependent manner, but did not affect the cell counts. The minimum effective concentration was 1 μM, and the EC_50_ was 7.68 ± 1.02 μM, which was lower than that required for HIF1 accumulation. Meanwhile, PAC-1 and BP also did not decreased HIF1α level. These results indicate that PAC-1 specifically inhibits the hydroxylation of HIF1α.

Next, we examined the impact of PAC-1 on HIF1α mRNA levels using qPCR. As shown in Fig. 3C, treatment with PAC-1 for up to 3 h did not affect HIF1α mRNA levels, whereas a 6-h treatment significantly decreased HIF1α levels. Thus, PAC-1 induces HIF1α accumulation by blocking the degradation process, rather than upregulating HIF1α expression.

Finally, to test the functional activity of the accumulated HIF1α, we examined the expression levels of HIF1α downstream target genes, i.e., *VEGF-1* and *EPO*, by qPCR. Similar to hypoxic conditions (1% O_2_) or the addition of the hypoxia-mimetic chemical BP, PAC-1 administration upregulated these two genes (Fig. 3D), suggesting that the PAC-1-induced HIF1α was functional. The upregulation of other HIF1α target genes was also observed (Supplement Fig. 1).

### PAC-1 induces the DNA damage response

**Fig. 4:**
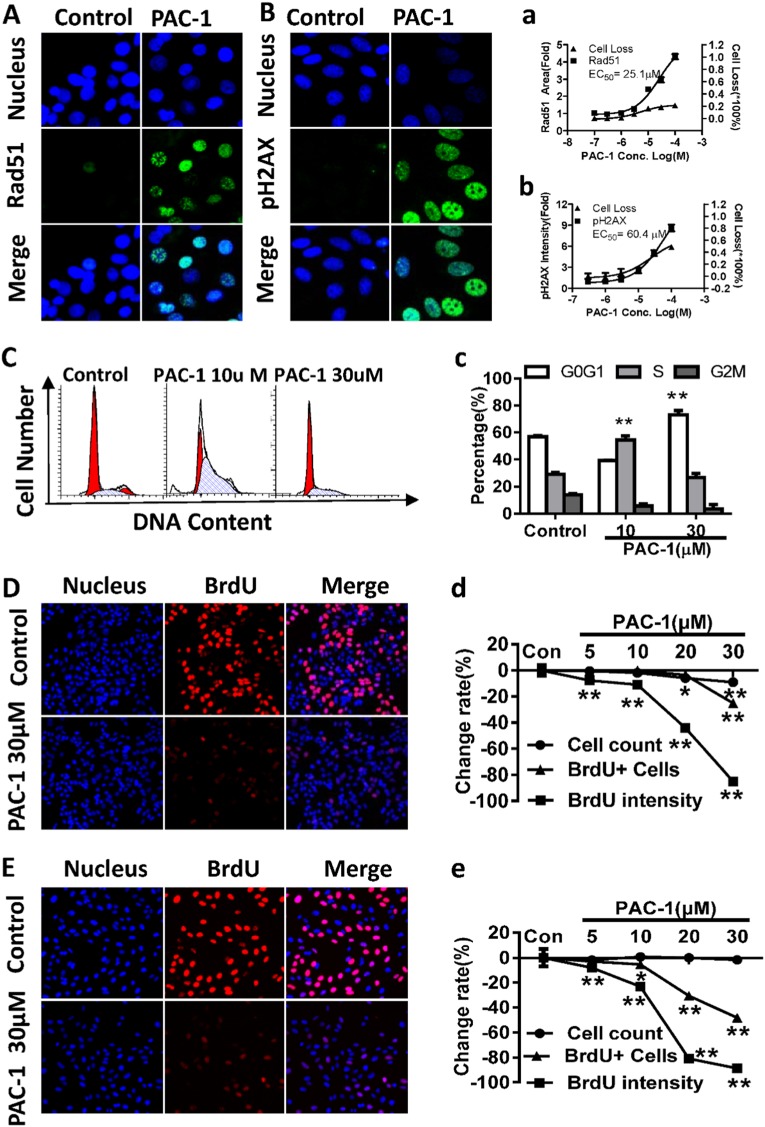
PAC-1 induces the DNA damage response, arrests cell cycle progression and blocks DNA synthesis. **A**, **a** Fluorescence images of RAD51 foci and concentration-dependent increases in the total areas of RAD51 foci after RAD51-EGFP_SW480 cells were treated with PAC-1 for 24 h. **B**, **b** Fluorescence images of accumulated pH2AX and the concentration-dependent increase in pH2AX intensity in HepG2 cells treated with PAC-1 for 24 h. **C**, **c** Cell cycle distribution in HepG2 cells after a 24-h PAC-1 treatment, as measured by flow cytometry. **D**, **E** BrdU incorporation status after PAC-1 exposure in HepG2 and U2OS cells. Cells were exposed to PAC-1 for 3 h before the addition of BrdU for another 3 h. Cells undergoing DNA synthesis within the last 3 h were stained with a BrdU-specific fluorescent dye (red). Images were generated by merging the DAPI and BrdU channels. **d**, **e** The EC_50_ was calculated using the data for BrdU intensity. Data represent means ± SEs of three independent experiments. **P < 0.01 and *P < 0. 05 compared with the corresponding control

The induction of RAD51 focus formation in RAD51-EGFP_SW480 cells following PAC-1 treatment was also observed in our screening assay. The RAD51 focus-inducing activity of 0.1–100 μM of PAC-1 was further assayed to confirm this finding. As shown in Fig. 4A, EGFP-labeled RAD51 foci formed in the nucleus in PAC-1-treated cells, whereas no foci formation was observed in the control group. A quantitative analysis of the fluorescence images for each treatment group revealed that the accumulation of RAD51 foci was concentration-dependent, with an EC_50_ of 25.1 μM (Fig. 4A). Additionally, the percent cell loss also increased in a concentration-dependent manner but remained below 25% (Fig. 4A). Because RAD51 is an important enzyme in DNA damage repair, the formation of RAD51 foci following a 24 h PAC-1 treatment indicated the presence of DNA damage.

pH2AX, another sensitive marker of DNA damage^15^, was also found to be induced by PAC-1 in HepG2 cells and two other cancer cell lines (Bxpc-3 and MCF-7 cells) after a 24 h treatment (Fig. 4B, Supplement Fig. 2). The quantitative analysis showed that the induction of pH2AX was also concentration-dependent (Fig. 4B, Supplement Fig. 2), with an EC_50_ of about 60.4 μM. Additionally, the cell count was decreased by about 40%. These results suggest that PAC-1 indeed induces DNA damage and cytotoxicity.

To investigate the contribution of caspases to cellular DNA damage, we treated RAD51-EGFP_SW480 cells with PAC-1 and the pan-caspase inhibitor z-VAD-FMK. No changes in the formation of RAD51 foci were observed (Supplement figure S3), suggesting that the PAC-1 induced RAD51 foci formation is not dependent on caspase activation.

### PAC-1 induces G_1_/S cell cycle arrest and blocks DNA synthesis in cancer cells

DNA damage is often accompanied by cell cycle arrest. The effects of PAC-1 on cell cycle progression were therefore examined using flow cytometry. We observed that after a 24 h treatment with 10 μM of PAC-1, the percentage of HepG2 cells in the S phase was significantly increased, whereas treatment with 30 μM of PAC-1 induced a significant G_0_/G_1_ phase arrest (Fig. 4C, c).

We then evaluated the effects of PAC-1 on DNA synthesis using BrdU incorporation assays in HepG2 and U2OS cells. A 3 h PAC-1 pre-exposure (5–100 μM) decreased BrdU incorporation in a concentration-dependent manner, as shown by the reduced number of BrdU-positive cells and the intensity of BrdU-specific fluorescence (Fig. 4D, E). The EC_50_ values were 20.49 ± 1.33 and 13.80 ± 0.68 μM for HepG2 and U2OS cells, respectively, but no reduction in the cell counts was observed (Fig. 4d, e). Thus, the inhibition of DNA synthesis by PAC-1 in both cell lines was independent of cytotoxicity.

### PAC-1 can sequester ferrous iron

**Fig. 5:**
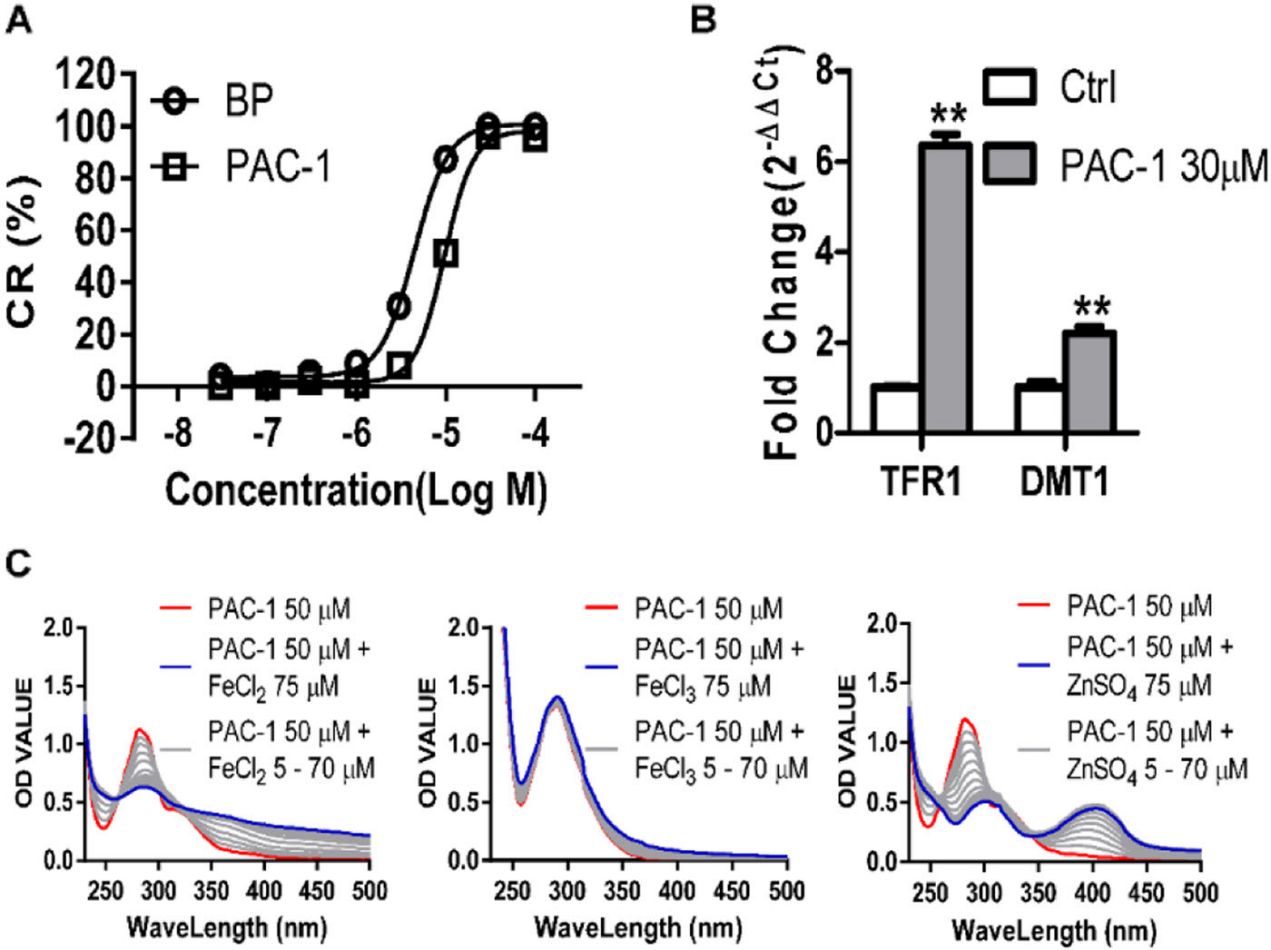
Iron sequestration potential of PAC-1. (**a**) Determination of the iron sequestration activity of PAC-1 and the iron chelator BP in ferrozine-iron-chelating assays. (**b**) Effects of PAC-1 treatment on TFR1 and DMT1 expression in HepG2 cells. (**c**) UV-Vis absorbance spectrum of changes induced by treatment with 50 μM PAC-1 and FeCl2 or ZnSO4 titration (steps set as 5 μM). Data represent means ± SEs of three independent experiments

The accumulation of unhydroxylated HIF1α after PAC-1 exposure suggested the inhibition of HIF1α hydroxylation, which is catalyzed by PHD2 and is an iron (II)-dependent process^16^. The high degree of similarity between the PAC-1 and BP HIF1α-induction profile (including its accumulation, hydroxylation status, and functional status), together with the finding that PAC-1 chelates zinc (II) led us to examine its iron (II) chelation ability. To address this, we performed ferrozine-iron (II) chelation assays^17^. Similar to the well-known iron (II) chelator BP, PAC-1 showed a concentration-dependent iron (II) chelating activity (Fig. 5a). The zinc-chelating potential of PAC-1 was evaluated by monitoring changes in the PAC-1 ultraviolet-visible (UV-Vis) absorbance spectra in buffer A (50 mM HEPES, 100 mM KNO_3_, pH 7.2) in response to ZnSO_4_ titration^9^. Using the same system, we found that the titration of FeCl_2_ (but not FeCl_3_) also changed the UV-Vis spectra of PAC-1 but that the effects of the FeCl_2_ titration were weaker than those of the ZnSO_4_ titration (Fig. 5c). Moreover, the mRNA levels for genes responsible for iron uptake, including transferrin receptor 1 (TFR1) and divalent metal transporter 1 (DMT1), were upregulated after 6 h of PAC-1 exposure (Fig. 5b), indicating that PAC-1 induces intracellular iron shortages.

### Iron (II) supplementation reverses DNA damage and the HIF1α stabilization induced by PAC-1

**Fig. 6:**
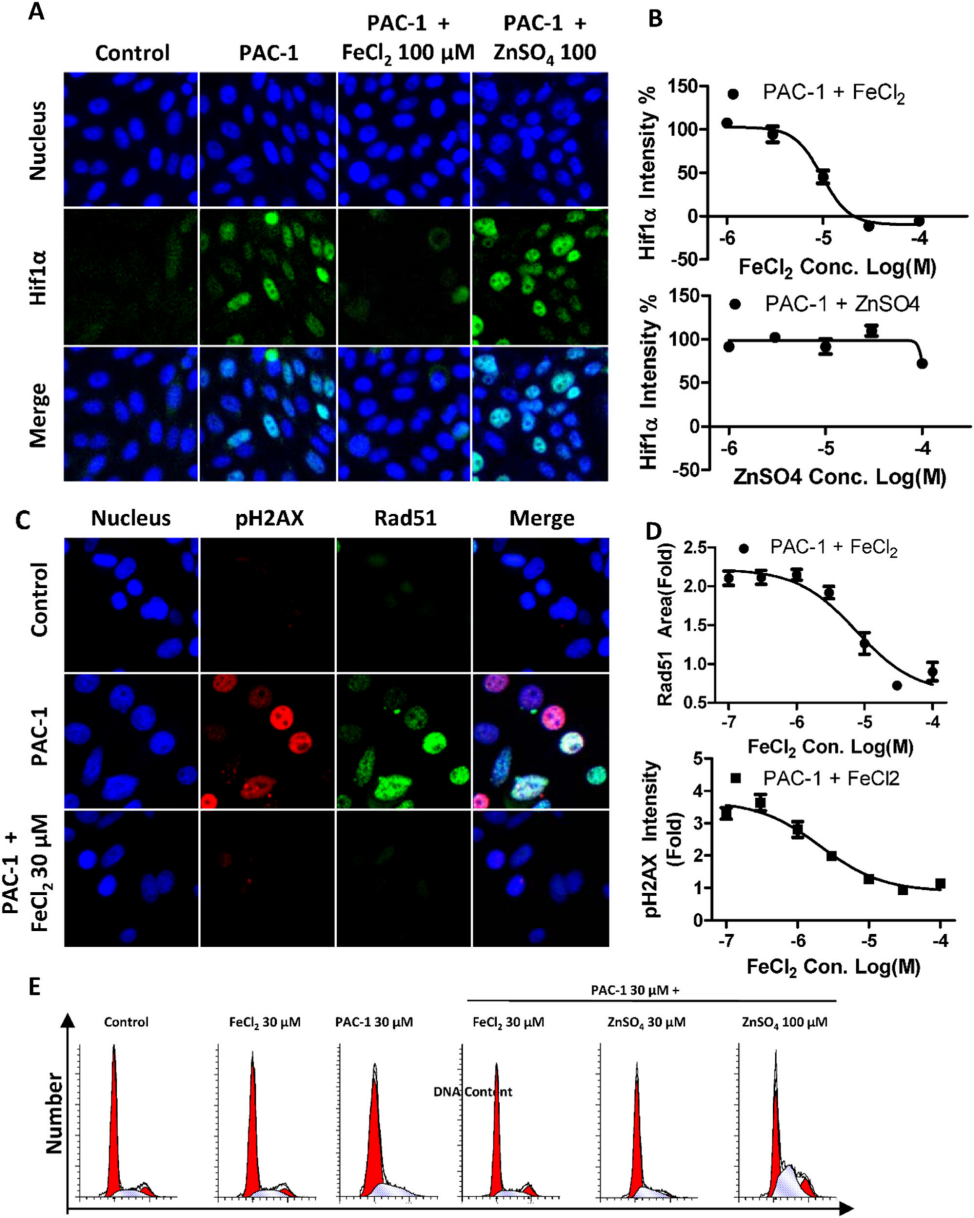
Iron supplementation reverses the HIF1α-stabilizing and DNA damage-inducing activities of PAC-1. **a**, **b** HIF1α stabilization status in HIF1α-EGFP_CHO cells after 3 h of treatment (PAC-1 concentration: 30 μM). **c**, **d** RAD51 and pH2AX levels in RAD51-EGFP_SW480 cells after 24 h of treatment with 30 μM PAC-1 with or without FeCl_2_ supplementation. **e** Cell cycle distribution status in HepG2 cells after the indicated treatments for 24 h. Data represent means ± SEs of three independent experiments

To confirm that iron sequestration by PAC-1 was indeed responsible for HIF1α stabilization, the effects of supplementation with additional iron were examined. HIF1α was found to be downregulated in a concentration-dependent manner with increasing concentrations of FeCl_2_, whereas zinc supplementation did not show similar effects (Fig. 6a, b). Moreover, the formation of RAD51 and pH2AX foci was reversed in a concentration-dependent manner by FeCl_2_ (Fig. 6c, d), and the effects of PAC-1 on the G_1_/S cell cycle arrest were also abolished by supplementation with iron (II), but not zinc (Fig. 6e). These results suggest that the specific cellular responses described above are all due to the cellular iron shortage induced by PAC-1.

### Iron (II) and hemin supplementation reverse the antiproliferative effects of PAC-1

**Fig. 7:**
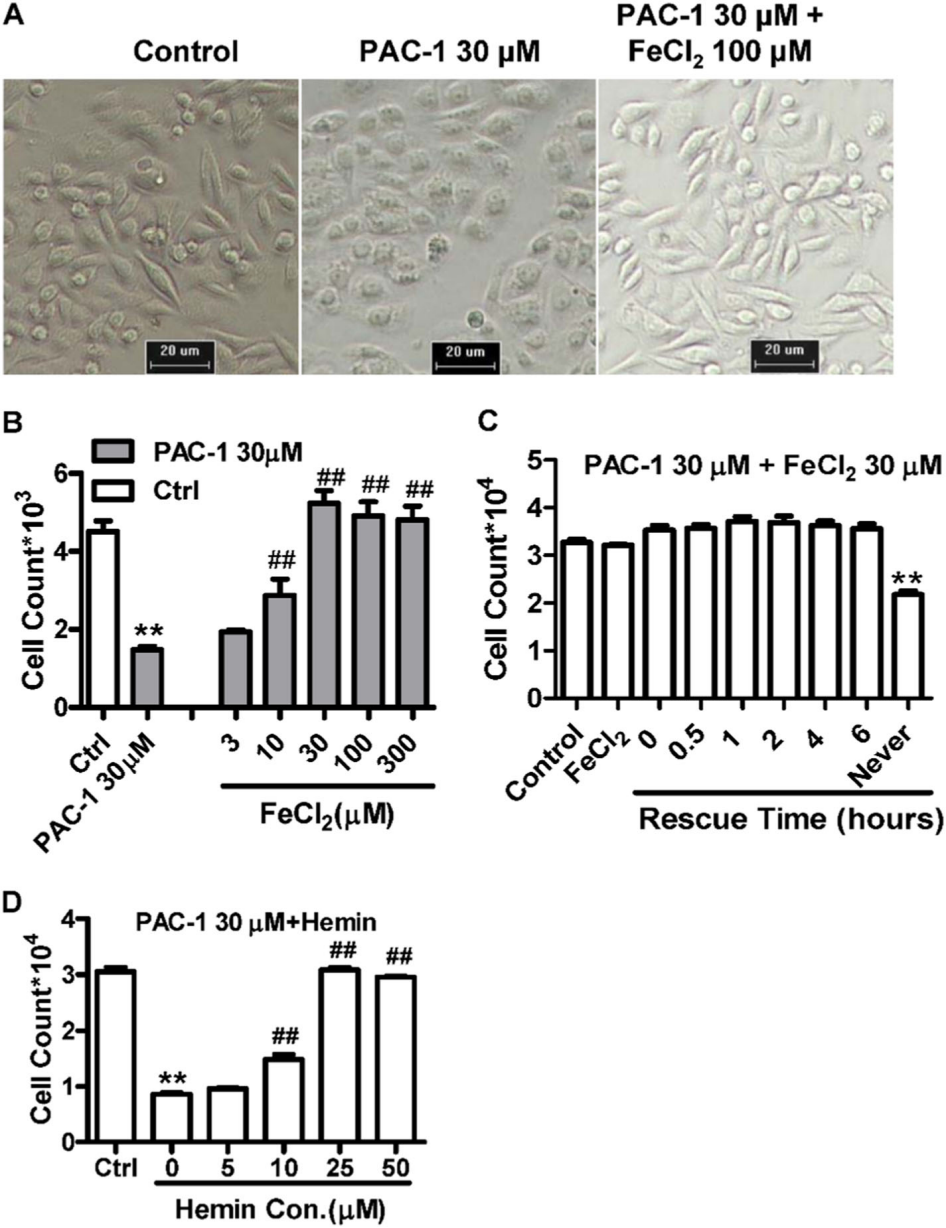
Iron supplementation (**a**–**c**) and treatment with the iron donor hemin (**d**) reverses the antiproliferative activity of PAC-1. **a** HepG2 cell morphology after treatment with the indicated compounds for 24 h. **b** Impact of FeCl_2_ on the PAC-1 antiproliferative activity. HepG2 cells were treated for 48 h before detection. **P < 0.01 compared with the control; ^##^*P* < 0.01 compared with the PAC-1 30 μM group. **c** Effects of different FeCl_2_ supplementation times on the PAC-1 antiproliferative activity. HepG2 cells were exposed to the indicated compounds and after various times, 30 μM FeCl_2_ was added. **d** The cells were exposed to compounds for 24 h before detection. Data represent the means ± SEs of three independent experiments. **P < 0.01 compared with the control. ##*P* < 0.01 compared with the PAC-1 30 μM group

To further confirm that the antitumor effects of PAC-1 were related to a cellular iron shortage, we also examined the effects of iron and an iron donor (hemin) on the antiproliferative activity of PAC-1. As shown in Fig. 7a, 24 h after the administration of PAC-1 changes in cell morphology were apparent, including bubble and granule formation. However, supplementation with 100 μM of FeCl_2_ completely rescued these changes in cell morphology and reversed the antiproliferative effects of PAC-1 in a concentration-dependent manner (Fig. 7b). Although hemin supplementation had similar effects, the effects of zinc supplementation were relatively weak compared with those of iron supplementation (Fig. 7d, Supplement Fig. 4). The same results were replicated in two other cancerous cell lines (Supplement Fig. 4). Moreover, iron rescue assays were performed by adding FeCl_2_ after PAC-1 exposure, thereby guaranteeing the entry of the compound into the cells. As shown in Fig. 7c, FeCl_2_ supplementation rescued the antiproliferative effects induced by the pre-exposure to PAC-1 for 6 h, confirming that iron does reverse the intracellular activity of PAC-1. Taken together, these results suggest that iron sequestration plays a key role in mediating the activity of PAC-1.

## Discussion

Off-target or multi-target effects are commonly observed for small-molecule drugs, and even for molecularly targeted drugs; therefore, a systematic screening of the activities of the lead or candidate compounds on multiple cellular pathways/targets is essential for drug discovery^18,19^. During a comprehensive screening assay performed in our laboratory, PAC-1 was found to specifically induce cellular responses, including the stabilization of HIF1α (under normoxic conditions) and the formation of RAD51 foci, in a concentration-dependent manner in EGFP-labeled reporter cell lines. Furthermore, the effects of PAC-1 on HIF1α stabilization and DNA damage were validated in HepG2 cells and two other cancer cell lines, and the underlying mechanism was revealed.

HIF1α is the main sensor and regulator of intracellular oxygen levels and undergoes hydroxylation, ubiquitination, and proteasome degradation after translation when oxygen levels are normal^20^. Low intracellular oxygen levels block HIF1α degradation, thereby leading to the accumulation of the protein inside the cell. The accumulating HIF1α then activates the transcription of genes responsible for angiogenesis, erythropoiesis, and glycolysis^21^. The hydroxylation of HIF1α is catalyzed by PHD2, an iron (II)- and 2-OG-dependent dioxygenase^22^. Iron (II) chelators, such as BP and deferrioxamine, are considered hypoxia mimetics because they induce HIF1α stabilization by sequestering iron (II) and therefore interfere with PHD2 activity^23^. In this study, a 3 h PAC-1 treatment induced the stabilization of HIF1α in a concentration-dependent manner both in HIF1α-EGFP_CHO cells and HepG2 cells but did not cause any evident cytotoxicity. These results clearly indicate that the HIF1α-stabilizing effects of PAC-1 represent a specific and early cellular event, which occurs prior to cellular damage or death. Moreover, similar to the effect of the iron (II) chelator BP, the HIF1α induced by PAC-1 was functional and unhydroxylated on Pro564. Moreover, HIF1α-OH formation following MG132 treatment was inhibited by PAC-1, which also upregulated HIF1α downstream target genes, including *VEGF* and *EPO*. Because PAC-1 is a potent inhibitor of HIF1α-OH formation and leads to changes in HIF1α kinetics, hydroxylation, and activity similar to BP, while iron supplementation reverses the HIF1α-stabilizing activity of PAC-1, we concluded that PAC-1 may induce HIF1α stabilization by sequestering iron and thus blocking HIF1α hydroxylation.

PAC-1 is a metal ion-chelating agent^24,25^. The core mechanism through which PAC-1 activates procaspase-3 involves zinc chelation. The zinc-chelating activity of PAC-1 can be attributed to the ortho-hydroxy *N*-acyl functionality in its structure^9,26^, which was commonly believed to chelate bivalent metal ions, such as iron, zinc, and copper^9,27,28^. Moreover, this functionality forms a tridentate O,N,O donor set (Supplement Fig. 5, red), which is frequently found in common bioactive iron chelators, including NIH^29^. Therefore, the notion that PAC-1 may chelate iron (II) is reasonable. Our ferrozine-iron (II) sequestration assays and the titration of FeCl_2_ (but not FeCl_3_) altered the UV-Vis absorbance spectra and support this hypothesis. Moreover, treatment with PAC-1 upregulated the levels of TFR1 and DMT1, two markers of intracellular iron shortages, in HepG2 cells. Importantly, iron chelators induce while zinc chelators contribute to HIF1α stabilization^30^. However, zinc chelation represses HIF1α transcription by preventing the binding of the HIF1 coactivator p300, thereby triggering the nuclear accumulation of nonfunctional HIF1α^30,31^. The upregulation of the VEGF and EPO HIF1α target genes following PAC-1 treatment indicated that iron rather than zinc sequestration plays a dominant role in the PAC-1-induced HIF1α accumulation.

The emergence of RAD51 foci was another important finding in our screening process. Because RAD51 foci are indicators of DNA damage, the DNA damage-inducing potential of PAC-1 was tested using another sensitive DNA damage marker, pH2AX. Interestingly, this marker also increased in a concentration-dependent manner following PAC-1 treatment, and was accompanied by cell death in HepG2, Bxpc-3, and MCF-7 cells. Furthermore, a DNA damage-related G_0_/G_1_/S phase cell cycle arrest was also observed in HepG2 cells. Because PAC-1 was first reported as a precaspase-3 agonist, we examined the contributions of caspases to DNA damage. The pan-caspase inhibitor z-VAD-FMK failed to block the DNA damage induced by PAC-1, indicating that caspase-3 activation was not the major contributor to the observed effects. Thus, our findings suggest that PAC-1 may be an iron (II) chelator and may inhibit the production of dNTPs in cancer cells by blocking the activity of ribonucleotide reductase (RNR). Indeed, RNR is also an iron-dependent enzyme and plays a critical role in regulating the total rate of DNA synthesis during cell division and DNA repair^32–34^, thereby inducing a G_1_/S cell cycle arrest^35^. Consistent with the above observations, we found that PAC-1 blocked cellular DNA synthesis and that iron supplementation reversed the DNA damage and cell cycle arrest induced by PAC-1. More importantly, the PAC-1 EC_50_ value for the inhibition of DNA synthesis was clearly lower than that for the induction of DNA damage. These results confirmed the cause and effect relationship between DNA damage and iron sequestration. Therefore, the emergence of PAC-1-induced DNA damage may also be due to its iron (II) sequestration ability.

Both zinc and iron are vital trace elements, which are involved in various cellular activities and are essential for normal cellular function^24,25^. As an antitumor candidate, the antiproliferative effects of PAC-1 on HepG2, MCF-7, and BxPC-3 cancer cells were observed following Fe^2+^ and Zn^2+^ supplementation. Interestingly, iron supplementation reversed the antiproliferative effects of PAC-1 in a concentration-dependent manner and rescued the antiproliferative effects resulting from a 6 h pre-exposure to PAC-1. Moreover, the stabilization of HIF1α and the G_1_/S cell cycle arrest were reversed by iron (II) rather than zinc supplementation, and the PAC-1-induced DNA damage was also reversed by iron supplementation. Therefore, iron (II) sequestration may play a more important role for PAC-1 activity than zinc chelation. These results could be explained by the observation that the labile iron pool, present in cells at about 1 μM^36^, is much higher than that of zinc, which is present in cells at 5–1000 pM^37^, even though the zinc-chelating ability of PAC-1 may be stronger at the molecular level. As described above, the hypoxic response induced by PAC-1 represents an early cellular stress response. While it does not elicit evident cytotoxicity at the time of detection, it can lead to oxidative damage and cause an imbalance in the mitochondrial membrane potential after 24 h of PAC-1 treatment (Supplement Fig. 6). Additionally, the PAC-1-induced DNA damage was accompanied by a significant decrease in cell counts. Furthermore, because RNR catalyzes the de novo synthesis of deoxyribonucleotides, which are precursors of DNA synthesis, it is essential for cell proliferation. Taken together, these results indicate that PAC-1 exerts its antiproliferative effects via the sequestration of cellular iron.

**Fig. 8:**
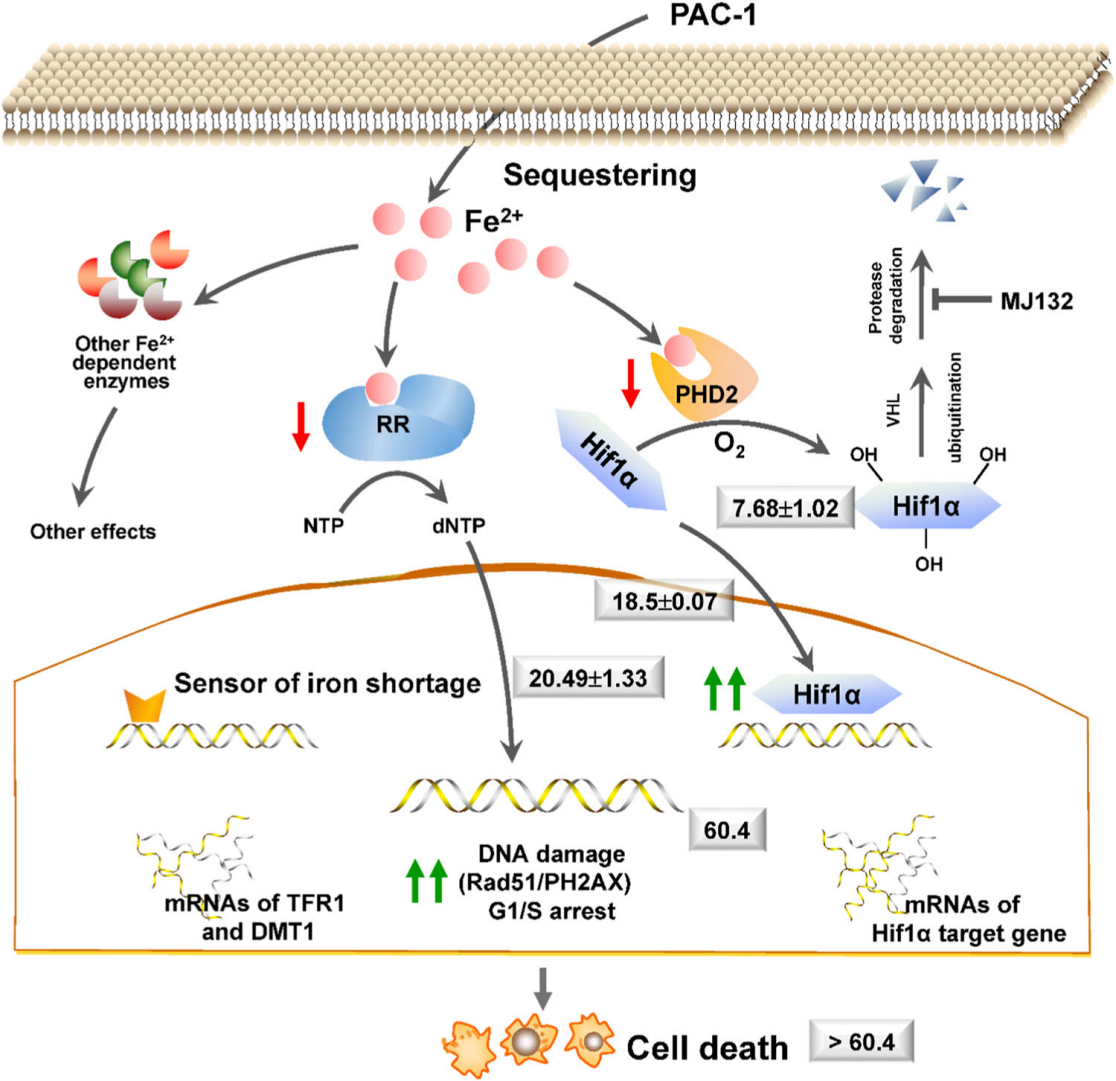
Summary of the regulatory mechanism underlying the PAC-1 induction of HIF1α accumulation and DNA damage

Iron chelators can block the activities of PHD2 and RNR, two well-known iron-dependent enzymes. A limitation of our study was the lack of availability of pure active protein, and thus the direct effects of PAC-1 on the activities of PHD2 and RNR were not addressed here. However, the functionally related cellular responses, including the inhibition of MG132-induced HIF1α-OH formation, HIF1α accumulation, DNA synthesis, DNA damage, and G_1_/S cell cycle arrest, which are commonly observed after the administration of iron chelators^23,32,33^, were all observed in PAC-1-treated cells. Additionally, the PAC-1 EC_50_ values for these responses gradually increased from upstream to downstream (Fig. 8). Moreover, the observation that supplementation with extra iron (II) abolished all of these effects further confirmed the cause–effect relationship between the iron-chelating and intracellular activities of PAC-1.

Taken together, our results showed for the first time that PAC-1 induces HIF1α stabilization, DNA damage, and cell cycle arrest in cancer cells. Mechanistic analyses confirmed that PAC-1 exerted these activities mainly via the sequestration of cellular iron. Our findings provide important insights into the intracellular activity of PAC-1 and may facilitate the development of related anticancer compounds.

## Electronic supplementary material

supplementary figure 1-6
