# Supplementary material for: Procaspase-3-activating compound 1 stabilizes hypoxia-inducible factor 1α and induces DNA damage by sequestering ferrous iron
Source: Cell Death Dis. 2018 Oct 4;9(10):1025. doi: 10.1038/s41419-018-1038-3 (PMC6172261; doi:10.1038/s41419-018-1038-3)
Supplement: Supplementary file 1 — supplementary figure 1-6 [file 41419_2018_1038_MOESM1_ESM.docx]

**Supplementary materials**

**

**

**Supplement figure 1. Upregulation of the *LDHA* and *PGK1* HIF1α downstream target genes following PAC-1 exposure.** Data represent means ± SEs of three independent experiments.


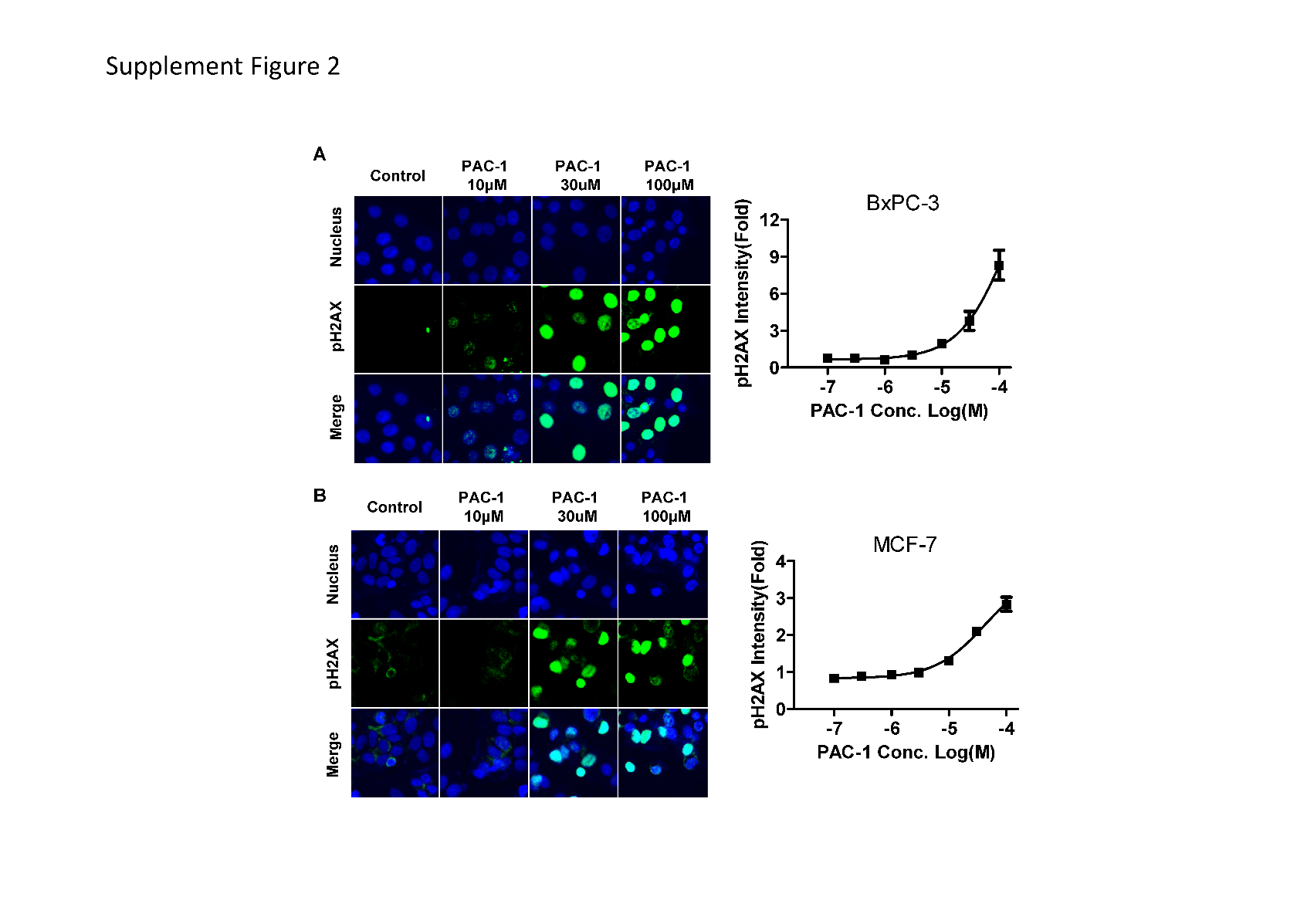


**Supplement figure 2. DNA-damage induction in Bxpc-3 cells (A) and MCF-7 cells (B) following PAC-1 treatment.** Cells were treated accordingly for 24 h before labelling with pH2AX-specific antibodies. Data represent means ± SEs of three independent experiments.


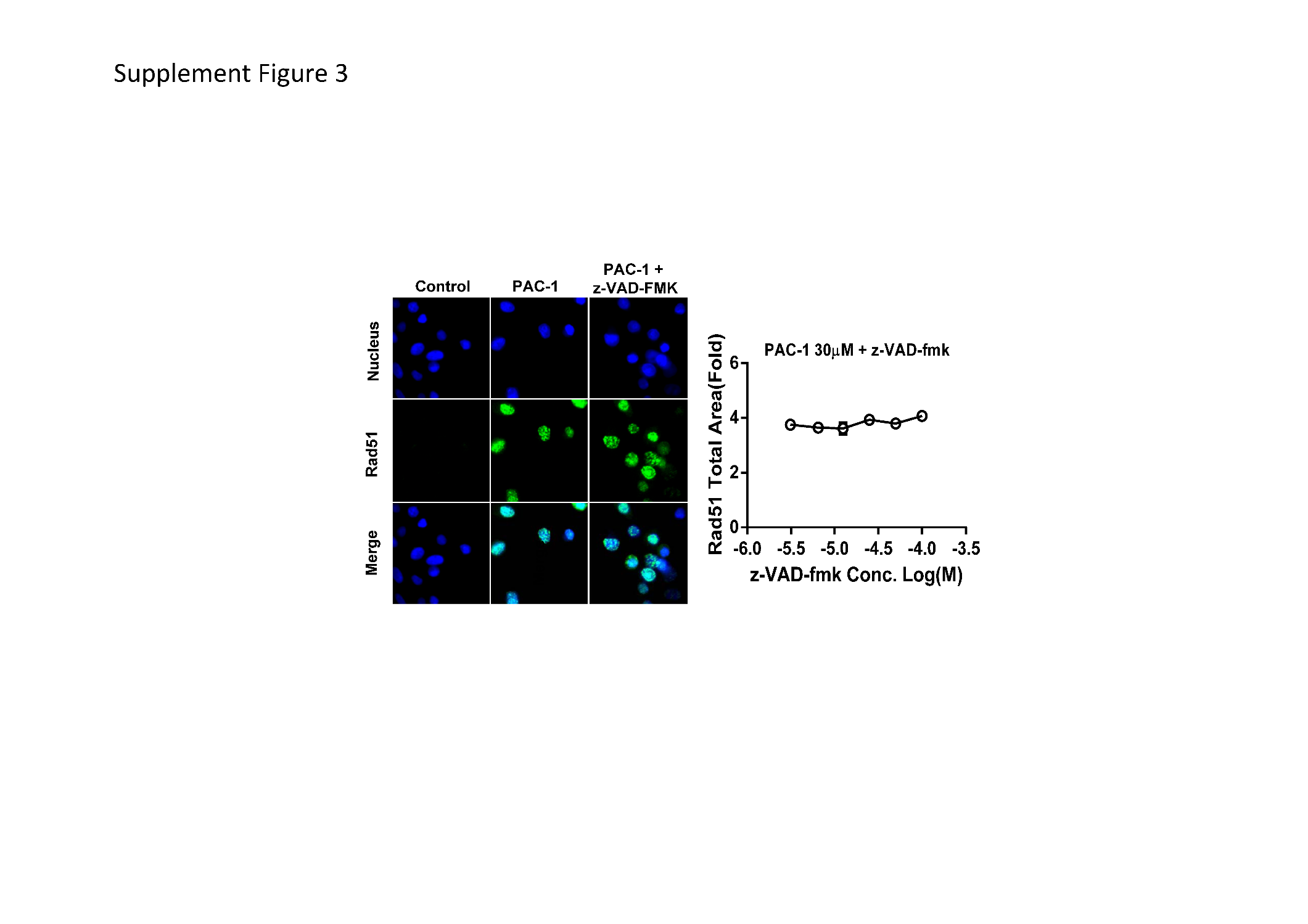


**Supplement figure 3. The pan-caspase inhibitor z-VAD-FMK does not affect the formation of RAD51 foci following PAC-1 treatment.** RAD51-EGFP_SW480 cells were exposed to 30 μM PAC-1 with or without increasing concentrations of z-VAD-FMK for 24 h before the detection of RAD51 fluorescence. Data represent means ± SEs of three independent experiments.


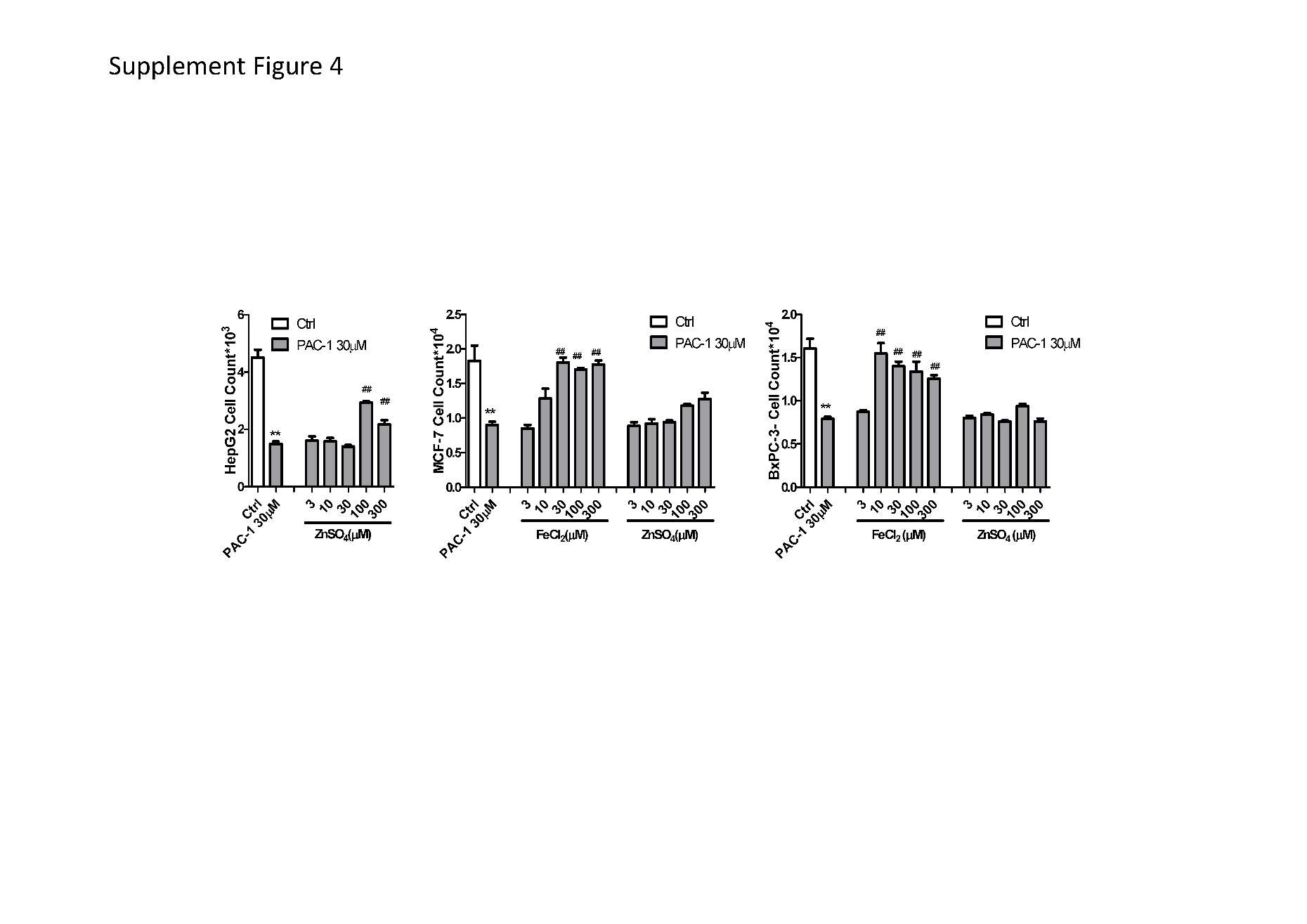


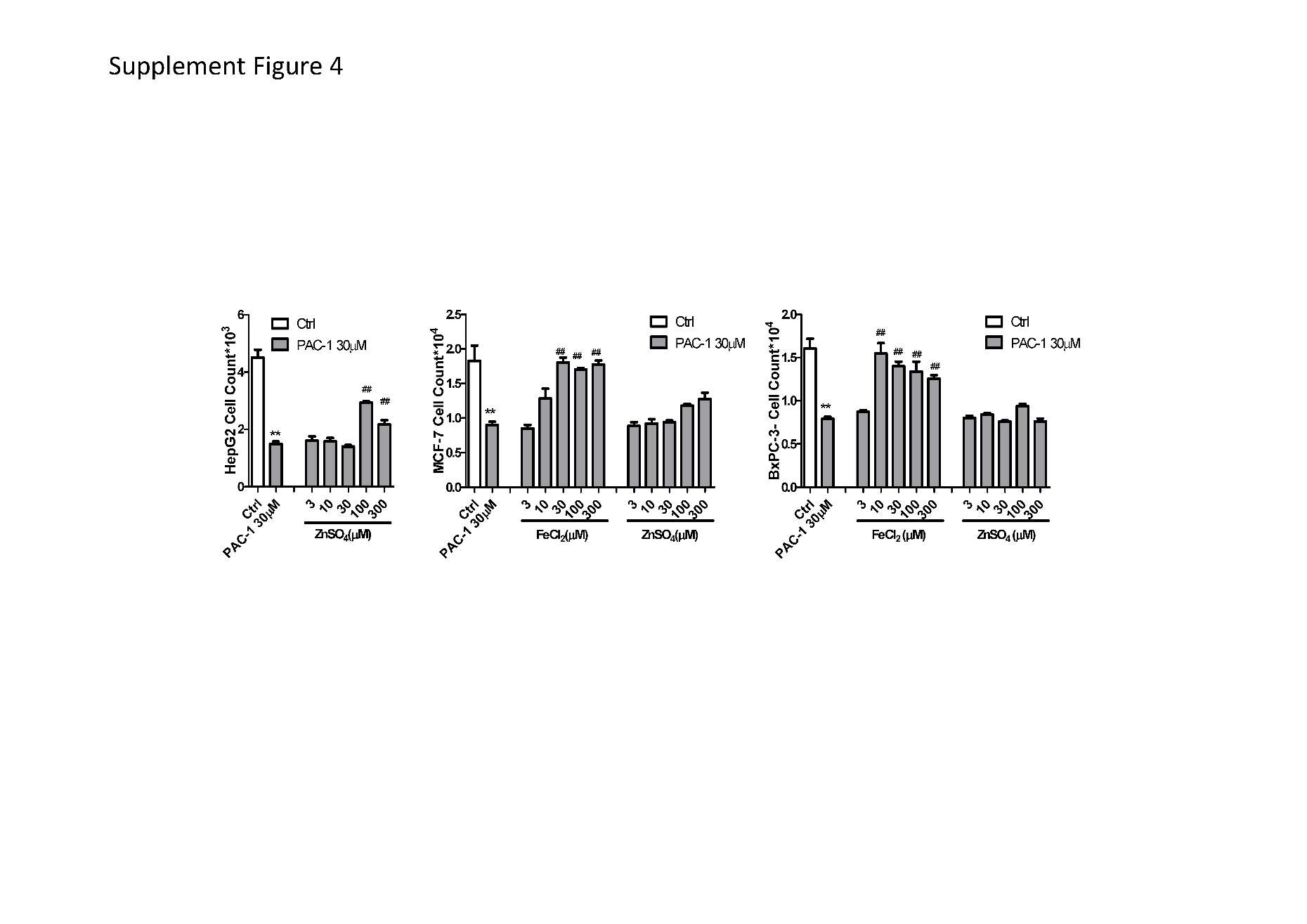


**Supplement figure 4. Comparison between the influence of iron and zinc supplementation on the PAC-1 antiproliferative activity.** Cells were treated accordingly for 48 h before detection. Data represent means ± SEs of three independent experiments. **, P < 0.01 compared with the control; ##, P < 0.01 compared with the PAC-1 30 μM treatment group.


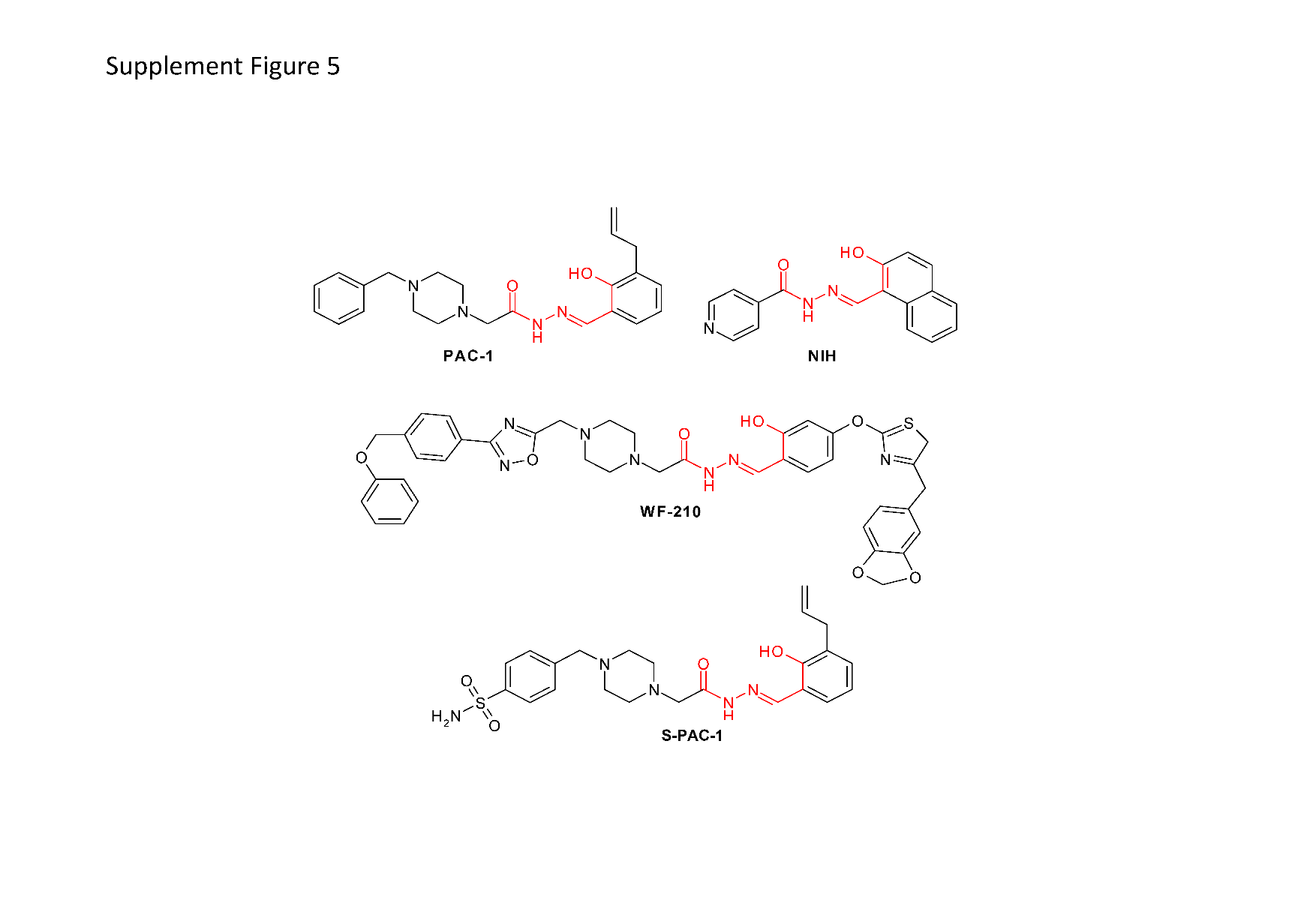


**Supplement figure 5. Chemical structures of the iron chelator NIH, PAC-1, and PAC-1 analogues.** The red line represents the motif responsible for zinc chelation by PAC-1 and the motif responsible for iron chelation by NIH.


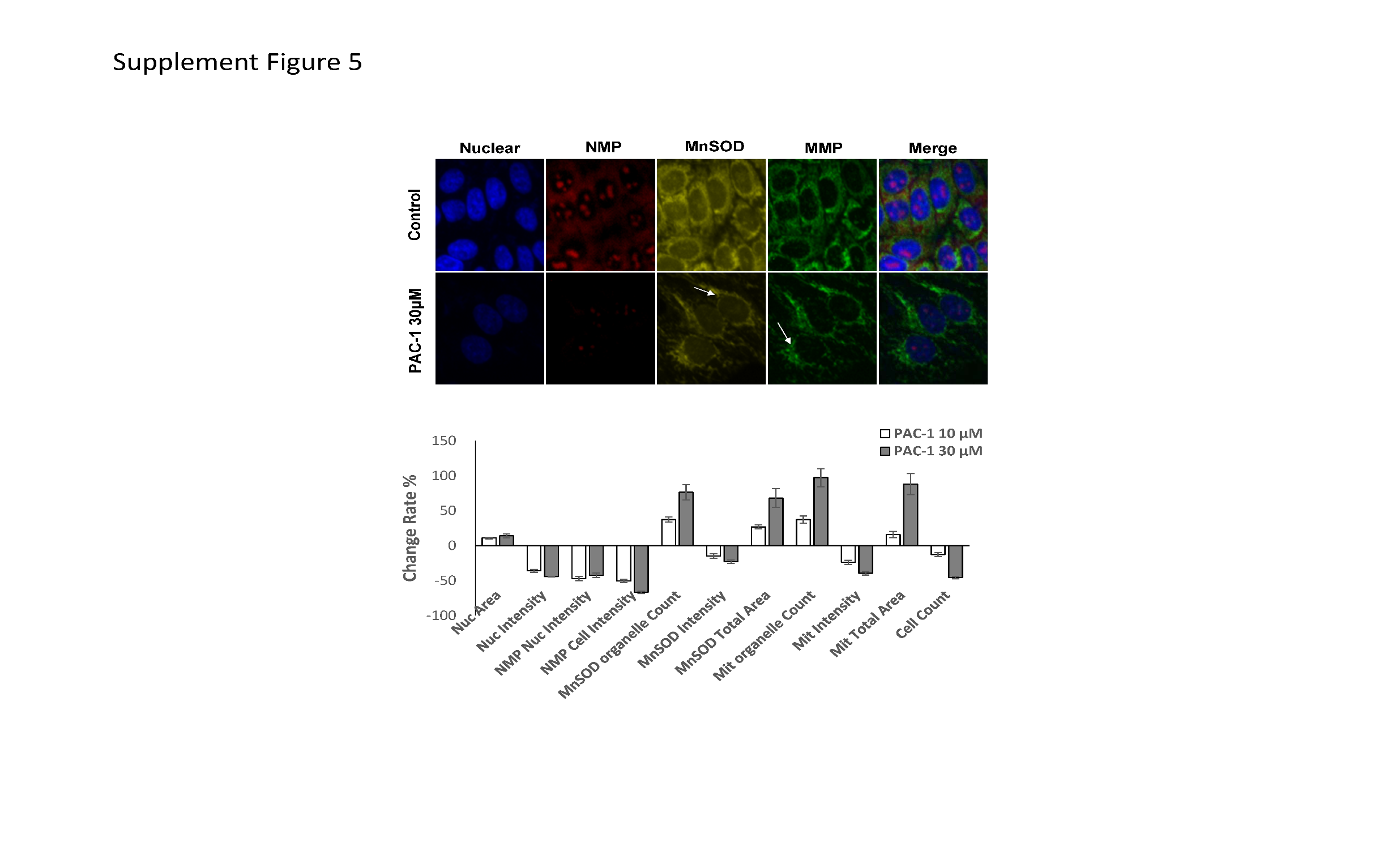


**Supplement figure 6. The effects of PAC-1 on multiple cytotoxic parameters using high-content analysis.** HepG2 cells were treated for 24 h before formaldehyde fixation, fluorescent labelling, image acquisition, and quantitative analysis of collected images. The fluorescence images were acquired with IN CELL Analyzer 2000 (GE Healthcare) under 20× objective lens. Nuclei (blue) were labelled with Hoechst 33342, MnSOD (yellow) was labelled with MnSOD-specific antibodies, mitochondria (green) were labelled with the mitochondrial membrane potential dye Mito-tracker Red, and Toto-3 was used as a cell membrane permeability indicator (red). Arrowheads indicate the formation of MnSOD granules and mitochondrial granules. Fifteen images for the same treatment were quantitatively analysed and normalised to the values in the control group; 1–4: nucleus, 5–8: Toto-3, 9-14: MnSOD, 19-20: mitochondria,20-22: cell count. Data represent means ± SEs of three independent experiments.
